# Organic–Inorganic Hybrid Gel Microspheres as a Plugging Agent for Ultra-High Temperature and High-Salinity Water-Based Drilling Fluids

**DOI:** 10.3390/gels12080733

**Published:** 2026-08-17

**Authors:** Yuanwei Sun, Jinsheng Sun, Kaihe Lv, Xianbin Huang, Jingping Liu

**Affiliations:** 1 State Key Laboratory of Deep Oil and Gas, China University of Petroleum—East China (UPC), Qingdao 266580, China; 2 School of Petroleum Engineering, China University of Petroleum—East China (UPC), Qingdao 266580, China

**Keywords:** plugging agent, water-based drilling fluid, ultra-high temperature and high-salinity, organic–inorganic hybrid gel microsphere

## Abstract

With the continuous expansion of ultra-deep and deep well drilling toward complex geological formations, the performance stability of water-based drilling fluids and wellbore stability under ultra-high temperature and high-salinity conditions have become critical challenges. High temperature and salt contamination can induce the degradation or failure of drilling fluid additives, while the development of pores and fractures in complex formations further increases the risk of filtrate invasion. Conventional polymer and inorganic plugging agents often suffer from insufficient thermal stability, poor salt tolerance, or limited adaptability to complex pore structures. In this study, an organic–inorganic hybrid gel microsphere plugging agent (HGP) with a core–shell structure was developed by in situ polymerization of AMPS, styrene (St), and sodium styrene sulfonate (SSS) on KH570-modified nano-SiO_2_. The hybrid microspheres consisted of a rigid SiO_2_ core and a flexible polymer shell, providing synergistic thermal stability, mechanical strength, and deformation capability. Structural characterization confirmed the successful formation of the designed organic–inorganic hybrid structure. After aging at 240 °C, HGP maintained stable morphology and dispersion characteristics, while exerting minimal influence on drilling fluid rheological properties. The addition of 3 wt% HGP reduced API fluid loss by approximately 30% and decreased sand bed invasion by approximately 50% after high-temperature aging. Under 35 wt% NaCl and 5 wt% CaCl_2_ contamination, HGP maintained effective filtration control, reducing fluid loss by more than 50% compared with the base fluid. Furthermore, HGP achieved core plugging efficiencies above 94% and reduced mud cake permeability by over 70%, demonstrating superior plugging performance compared with polymer microspheres NF-1 and SiO_2_ particles. The enhanced performance was considered to arise from the synergistic effects of stable dispersion, pore-throat bridging, deformation filling, and structural stabilization. This study provides a rigid–flexible hybrid strategy for designing high-performance plugging agents for ultra-high temperature and high-salinity water-based drilling fluids.

## 1. Introduction

With the gradual depletion of conventional oil and gas resources and the increasing global energy demand, oil and gas exploration and development are progressively extending toward ultra-deep formations, deep-sea environments, and complex geological regions [1]. During the drilling of ultra-deep and deep wells, drilling fluid systems are commonly subjected to temperatures exceeding 200 °C, complex saline invasion, and high differential pressure conditions [2]. These extreme environments impose increasingly stringent requirements on the thermal stability, salt contamination resistance, and wellbore stability of water-based drilling fluids [3]. Under high-temperature conditions, polymeric additives in drilling fluids are prone to molecular chain scission, functional group degradation, and conformational changes, resulting in deteriorated rheological properties and reduced filtration control capability. Meanwhile, the invasion of high-concentration salt ions from formations can compress the electrical double layer of clay particles, leading to flocculation, sedimentation, and performance degradation of drilling fluid systems [4]. Furthermore, ultra-deep formations generally exhibit strong heterogeneity, with abundant natural fractures, induced fractures, and micro-/nano-scale pores. Driven by positive differential pressure, drilling fluid filtrate can readily invade formations through these channels, causing complex engineering problems such as formation damage, hole enlargement, and even wellbore instability. Therefore, constructing stable and effective plugging barriers to reduce filtrate invasion depth and maintain wellbore integrity while preserving fundamental drilling fluid properties is essential for safe and efficient drilling of ultra-deep wells. As a key functional material in water-based drilling fluid systems, plugging agents mainly form low-permeability plugging layers in the near-wellbore region through particle bridging, filling, and compaction, thereby reducing the invasion of drilling fluids into formation pores and fractures. An ideal plugging agent should possess an appropriate particle size distribution to effectively match pore throats with different sizes. Meanwhile, it should also exhibit excellent thermal stability, salt tolerance, and certain deformation and filling capability to adapt to complex formation environments. However, under ultra-high temperature and high-salinity conditions, plugging agents must not only overcome structural instability but also maintain good compatibility with drilling fluid systems to avoid adverse effects on rheological properties after addition [5]. Therefore, the development of novel plugging materials with high-temperature stability, environmental adaptability under saline conditions, and multi-scale plugging capability has become a critical strategy for improving the performance of ultra-high temperature and high-salinity water-based drilling fluids.

Currently, various types of plugging materials have been developed for complex high-temperature and high-salinity drilling environments, mainly including organic polymer-based plugging agents and inorganic particle-based plugging agents. Organic polymer-based plugging agents, such as crosslinked polymer microspheres [6], pre-crosslinked gel particles [7], and polymer nanomaterials [8], have attracted extensive attention due to their excellent flexibility, elasticity, and deformability. These materials can enter formation pores and fractures under differential pressure and achieve plugging through particle deformation, bridging, and filling. In particular, polymer gel-based materials can further enhance plugging performance through water absorption and swelling, making them widely investigated for formation sealing applications. However, conventional organic polymer materials generally suffer from limited temperature resistance. Under prolonged exposure to high temperatures, polymer chains are susceptible to backbone scission, destruction of crosslinked networks, and performance deterioration [9]. Meanwhile, high concentrations of salt ions can weaken the interactions between polymer chains, reduce particle stability, and even induce microsphere aggregation, thereby affecting their deep placement capability and effective plugging performance [10]. In contrast, inorganic particle-based plugging agents, such as SiO_2_, clay minerals, and metal oxide nanoparticles, exhibit good thermal stability, chemical inertness, and mechanical strength [11]. These materials can maintain structural integrity under ultra-high temperature conditions and form mechanically robust plugging structures. However, inorganic particles generally possess highly rigid structures with limited deformability, making them difficult to adapt to irregular pores and complex fracture networks [12]. Moreover, the plugging performance of single-component inorganic particles mainly relies on particle size matching. When formation pore size distributions are broad, plugging blind zones are easily generated, resulting in insufficient long-term plugging effectiveness. In addition, micro- and nanoscale inorganic particles are prone to aggregation under high-salinity conditions, leading to poor dispersion stability in ultra-high temperature and high-salinity drilling fluids and limiting their practical effectiveness [13]. Recently, organic–inorganic hybrid materials have emerged as promising candidates for high-performance plugging applications because they integrate the flexible adaptability of organic polymers with the high strength and thermal resistance of inorganic components [14]. However, existing organic–inorganic composite plugging agents still face several challenges, including insufficient interfacial bonding stability between organic and inorganic phases, deteriorated dispersion performance under high-salinity conditions, and difficulty in maintaining stable plugging structures during long-term exposure to high temperatures [15]. Therefore, constructing organic–inorganic hybrid plugging materials with stable interfacial structures, excellent environmental adaptability, and rigid–flexible synergistic plugging capability is critical for meeting the requirements of ultra-high temperature and high-salinity water-based drilling fluids.

Based on the above considerations, this study proposes a design strategy for an organic–inorganic hybrid gel microsphere plugging agent (HGP) to overcome the limitations of conventional polymer microspheres and inorganic particle-based plugging agents under ultra-high temperature and high-salinity conditions. KH570-modified nano-SiO_2_ was employed as an inorganic reinforcing component, and a polymer gel shell was in situ constructed on the SiO_2_ surface through copolymerization of 2-acrylamido-2-methylpropanesulfonic acid (AMPS), styrene (St), and sodium styrene sulfonate (SSS), forming a core–shell organic–inorganic hybrid gel microsphere structure with a “rigid SiO_2_ core–flexible polymer shell” architecture. In this structure, the SiO_2_ core and benzene ring structures jointly enhance the thermal stability and structural strength of the microspheres, while sulfonic groups provide salt resistance and dispersion stability. Meanwhile, the polymer gel shell endows HGP with flexible deformation capability, enabling adaptation to complex pore structures and effective plugging. The structural composition and thermal stability of HGP were systematically investigated using FTIR, XPS, ^1^H NMR, TG/DTG, SEM, and TEM analyses. Furthermore, the effects of HGP on the rheological properties, filtration control capability, and tolerance to salt and calcium contamination of ultra-high temperature and high-salinity water-based drilling fluids were evaluated. Multi-scale plugging experiments were further conducted to investigate the plugging performance of HGP toward pores and fractures with different sizes and to elucidate its plugging mechanism under ultra-high temperature and high-salinity conditions. This study provides a new strategy for the structural design of high-performance plugging agents for drilling fluids under extreme geological conditions and is expected to provide theoretical insights and technical support for improving wellbore stability during ultra-deep well drilling.

## 2. Results and Discussion

### 2.1. Characterization of HGP

#### 2.1.1. FTIR Analysis

To verify the chemical structure of the organic–inorganic hybrid gel microsphere plugging agent HGP and the interactions among its components, Fourier transform infrared spectroscopy (FTIR) analysis was performed.

**Figure 1:**
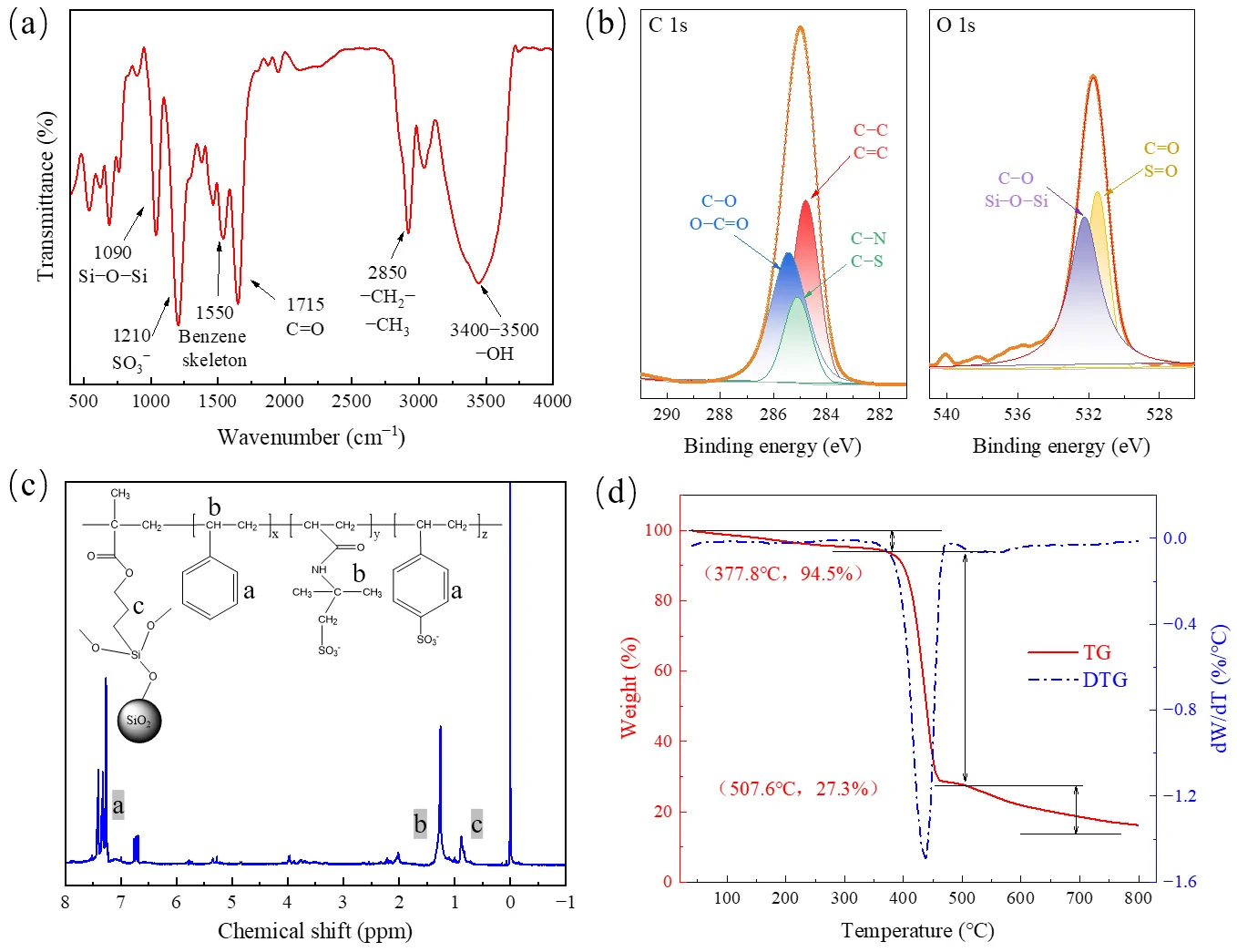
Characterization of HGP: (**a**) FTIR spectrum, (**b**) XPS C1s and O1s spectra, (**c**) ^1^H NMR spectrum, (**d**) TG/DTG curve.

Figure 1a presents the FTIR spectrum of HGP. A broad characteristic band around 3400 cm^−1^ is attributed to the stretching vibration of hydroxyl groups (–OH), mainly originating from the silanol groups on the surface of nano-SiO_2_ and adsorbed water molecules in the sample. Since the surface hydroxyl groups of nano-SiO_2_ cannot be completely consumed during the silanization reaction, and the sulfonic groups in 2-acrylamido-2-methylpropanesulfonic acid (AMPS) and sodium styrene sulfonate (SSS) exhibit strong hydrophilicity, this characteristic band remains observable [16]. The characteristic band near 2850 cm^−1^ corresponds to the stretching vibration of aliphatic C–H bonds, which mainly originates from the propyl chain of KH570 and the –CH_2_– groups in the polymer backbone [17]. This indicates the successful incorporation of silane coupling structures and organic polymer segments into the hybrid microspheres. The band at approximately 1715 cm^−1^ is assigned to the stretching vibration of carbonyl groups (C=O) in the methacrylate moiety of KH570. A characteristic band around 1550 cm^−1^ is associated with the stretching vibration of aromatic C=C bonds and aromatic ring skeletal vibrations, mainly derived from the styrene (St) and SSS structural units, confirming the participation of styrene-based monomers in the copolymerization process. The characteristic bands located in the range of 1180–1040 cm^−1^ are mainly attributed to the asymmetric and symmetric stretching vibrations of S=O bonds in sulfonic groups (–SO_3_^−^), originating from the sulfonic acid groups of AMPS and SSS. In addition, the band observed at approximately 1100 cm^−1^ corresponds to the asymmetric stretching vibration of Si–O–Si bonds, representing the silica framework [18]. Overall, the FTIR results demonstrate the coexistence of the SiO_2_ inorganic framework, KH570 silane coupling structures, and characteristic functional groups of the AMPS/SSS/St copolymer within HGP. These findings preliminarily indicate the effective incorporation of KH570-modified nano-SiO_2_ with the AMPS/SSS/St copolymer, leading to the formation of the designed organic–inorganic hybrid microsphere structure.

#### 2.1.2. XPS Analysis

To further investigate the surface elemental chemical states and functional group compositions of HGP, X-ray photoelectron spectroscopy (XPS) analysis was performed. The C 1s and O 1s spectra are presented in Figure 1b. Since XPS is a surface-sensitive characterization technique with a typical detection depth of only several nanometers, the obtained results mainly reflect the chemical composition of the polymer shell exposed on the microsphere surface.

The C 1s spectrum reveals that carbon elements on the surface of HGP exist in three different chemical environments. The characteristic peak located at approximately 284.8 eV is assigned to C–C/C=C bonds, mainly originating from the aromatic ring structures of St and SSS as well as the carbon backbone of the polymer chains, indicating the successful incorporation of styrene-based monomers into the polymer structure [19]. The peak at around 285.5 eV can be attributed to C–N/C–S-related carbon species. Specifically, the C–N component mainly originates from the amide structure of AMPS, while the C–S component is associated with carbon atoms linked to sulfonic groups in AMPS and SSS. The characteristic peak near 286.0 eV is assigned to C–O/O–C=O bonds. The C–O contribution is related to the ester groups of KH570 and oxygen-containing carbon structures introduced by the silane coupling agent, whereas the O–C=O component originates from the ester carbonyl groups in KH570. These results further confirm the involvement of KH570 in the construction of the hybrid microsphere structure [20].

After peak deconvolution, the O 1s spectrum can be fitted into two components. The lower binding energy component is mainly associated with oxygen species from sulfonic groups (–SO_3_^−^) and carbonyl groups, originating from oxygen-containing functional groups in AMPS, SSS, and KH570. The higher binding energy component is primarily attributed to oxygen in C–O bonds and may also include contributions from oxygen in Si–O–C structures, suggesting that KH570 effectively mediates the interfacial connection between the organic polymer phase and inorganic nano-SiO_2_ component [21].

Overall, the XPS results demonstrate that the surface of HGP contains abundant aromatic carbon structures, amide groups, sulfonic groups, and ester groups, confirming the successful incorporation of AMPS, St, SSS, and KH570 into the hybrid microsphere structure. Combined with the FTIR results, these findings further verify the successful construction of an organic–inorganic hybrid interface between KH570-modified nano-SiO_2_ and the AMPS/St/SSS copolymer.

#### 2.1.3. ^1^H NMR Analysis

To further verify the structural composition of the organic polymer shell in HGP and the incorporation of different functional monomers, proton nuclear magnetic resonance (^1^H NMR) spectroscopy was performed, and the spectrum is presented in Figure 1c. Since HGP theoretically possesses an organic–inorganic core–shell structure, the SiO_2_ core does not generate obvious ^1^H NMR signals. Therefore, the obtained spectrum mainly reflects the proton environments of the AMPS/St/SSS copolymer shell and the incorporated KH570-derived organic segments.

A distinct group of aromatic proton absorption signals (peak a) is observed in the chemical shift region of δ = 6.5–7.5 ppm [22]. These signals are mainly assigned to the aromatic protons of the benzene rings in the St and SSS structural units, confirming the successful incorporation of aromatic monomers into the copolymer network [23]. A broad signal (peak b) appears in the range of δ = 1.0–2.2 ppm, which is mainly attributed to aliphatic protons in the polymer chains. Specifically, the signals around δ = 1.2–2.0 ppm are mainly associated with –CH_2_– and –CH– protons in the polymer backbone, with additional contributions from methylene groups in the propyl chain of KH570. Notably, the absorption signals around δ = 1.8–2.2 ppm can be partially assigned to the gem-dimethyl protons [–C(CH_3_)_2_–] in AMPS structural units. After polymerization, AMPS units exist in different chain microenvironments, causing overlap between these signals and the aliphatic proton signals of the polymer backbone and resulting in peak broadening. The presence of this signal further confirms the incorporation of AMPS units into the copolymer structure. An absorption signal (peak c) located at δ = 0.8–1.0 ppm is observed, which mainly originates from the propyl bridge protons of the KH570-derived segments, especially the methylene protons adjacent to silicon atoms (–CH_2_–Si–) [24]. During the modification process, the siloxane end of KH570 undergoes hydrolysis and condensation with the surface of nano-SiO_2_, while the methacrylate group participates in free-radical polymerization. Therefore, the aliphatic chain segments of KH570 remain within the hybrid microsphere structure. The presence of this signal indicates that KH570 not only participates in the surface modification of SiO_2_ but also remains incorporated within the organic polymer network, acting as a linkage between the inorganic core and organic shell [25].

Furthermore, no obvious vinyl proton absorption signals are observed in the range of δ = 5.5–6.5 ppm. For AMPS, St, and KH570-derived methacrylate groups, this region corresponds to protons associated with C=C double bonds. The disappearance of these characteristic signals indicates that the functional monomers underwent polymerization, forming a stable polymer framework [26]. Overall, the ^1^H NMR spectrum exhibits characteristic signals corresponding to aromatic ring protons, AMPS-derived aliphatic protons, and KH570-derived propyl chain protons, while the disappearance of vinyl proton signals confirms the successful incorporation and polymerization of AMPS, St, SSS, and KH570 segments. Combined with the FTIR and XPS results, these findings further demonstrate that HGP possesses the expected core–shell structure consisting of a modified SiO_2_ core and a functional polymer shell.

#### 2.1.4. TG/DTG Analysis

To evaluate the thermal stability of HGP, thermogravimetric (TG) and derivative thermogravimetric (DTG) analyses were performed. As shown in Figure 1d, HGP exhibits a distinct multi-stage thermal weight loss behavior. From room temperature to 300 °C, only a slight mass loss of approximately 5% is observed, which is mainly attributed to the removal of physically adsorbed water and bound water associated with hydroxyl groups on the SiO_2_ surface. When the temperature increases above 370 °C, the TG curve exhibits a rapid decrease, and the DTG curve reaches the maximum degradation peak at 377.8 °C. This stage corresponds to the thermal degradation of the organic polymer shell, involving the decomposition of AMPS-, SSS-, and styrene-based copolymer segments as well as KH570-derived organic chains. Benefiting from the rigid aromatic structures, interfacial interactions between sulfonic groups and SiO_2_, and the confinement effect of the inorganic nano-SiO_2_ core, HGP exhibits enhanced thermal stability. With further increasing temperature, the TG curve gradually becomes stable, and approximately 16% of the initial mass remains at 800 °C, which is mainly attributed to the thermally stable SiO_2_ inorganic core. These results demonstrate that HGP possesses good thermal stability, and the multi-stage thermal degradation behavior reflects its characteristic organic–inorganic hybrid structure.

#### 2.1.5. Microscopic Morphology Analysis

**Figure 2:**
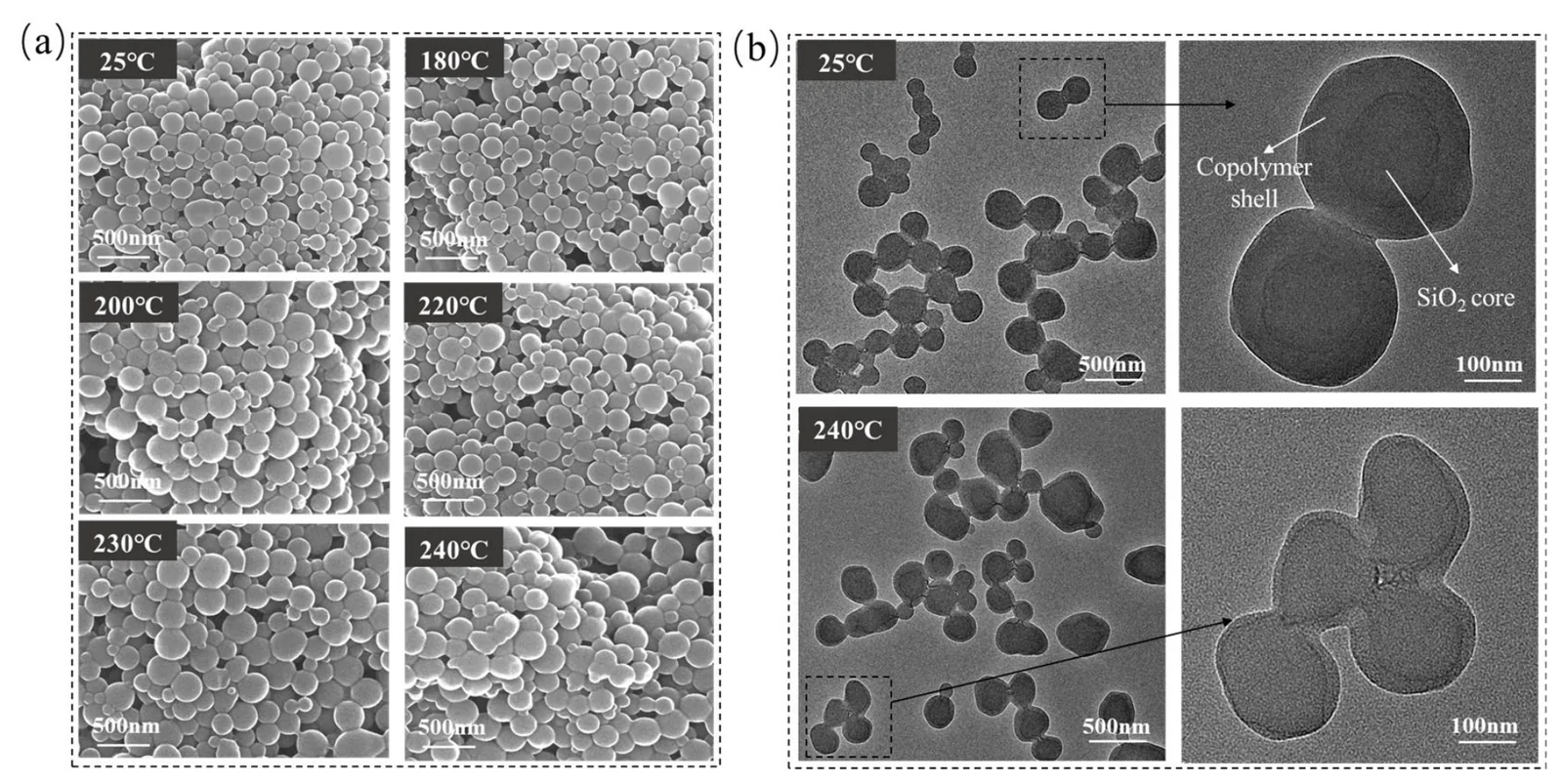
Microscopic morphology of HGP: (**a**) SEM images after aging at different temperatures, (**b**) TEM images before and after aging at 240 °C.

To evaluate the morphological stability of HGP under ultra-high temperature conditions, a 3 wt% aqueous dispersion of HGP was aged at 25, 180, 200, 220, 230, and 240 °C, followed by scanning electron microscopy (SEM) observation. The corresponding SEM images are shown in Figure 2a. At 25 °C, HGP exhibits a well-defined spherical morphology with a relatively uniform particle size distribution, mainly ranging from 150 to 300 nm. The microsphere surfaces are smooth, and no obvious aggregation, collapse, or fragmentation is observed, indicating that the core–shell hybrid microspheres with intact morphology were successfully synthesized through free-radical copolymerization of AMPS, St, and SSS using KH570-modified nano-SiO_2_ as the core component. As the aging temperature increases from 180 to 220 °C, the overall morphology of HGP remains highly stable, maintaining good sphericity and relatively uniform particle dimensions. Only slight particle contact and localized aggregation are observed. These results indicate that the polymer shell retains sufficient structural stability under high-temperature conditions. When the aging temperature is further increased to 230 and 240 °C, HGP still maintains a complete spherical profile without obvious melting, cracking, or collapse. A certain degree of particle adhesion and aggregation occurs, which is mainly attributed to the enhanced mobility of polymer chains and the increased probability of interparticle contact at elevated temperatures, rather than structural instability or degradation of the microspheres. Combined with the TG results, the main thermal degradation temperature of HGP is approximately 378 °C, which is significantly higher than the aging temperature of 240 °C. Therefore, the polymer framework remains largely intact during high-temperature aging, enabling HGP to maintain good particle integrity.

To further investigate the microstructure of HGP, transmission electron microscopy (TEM) characterization was conducted before and after aging at 240 °C, as shown in Figure 2b. Before aging, HGP exhibits a distinct core–shell structure. The darker region inside the microspheres corresponds to the nano-SiO_2_ core, while the lighter outer region is attributed to the AMPS/St/SSS copolymer shell. A clear interface between the core and shell can be observed, indicating that KH570 effectively facilitates the interfacial connection between inorganic SiO_2_ and the polymer phase, resulting in the formation of a stable organic–inorganic hybrid core–shell structure. After aging at 240 °C, the core–shell structure remains clearly visible. The SiO_2_ core maintains its integrity, and the polymer shell remains continuously coated around the core surface without obvious shell detachment, fracture, or exposure of the inorganic core. Although partial particle contact and aggregation occur after high-temperature aging, the core–shell architecture of individual microspheres remains intact, demonstrating that the organic–inorganic interfacial structure is not significantly disrupted under ultra-high temperature conditions.

Combined with the chemical structure analyses from FTIR, XPS, and ^1^H NMR, as well as the thermal stability evaluation from TG/DTG, these results confirm that HGP possesses the expected hybrid core–shell architecture. Moreover, HGP exhibits excellent physical and chemical stability under high-temperature conditions, maintaining structural integrity even after aging at 240 °C. These characteristics suggest that HGP has great potential for stable application and effective plugging performance in ultra-high temperature drilling fluid systems.

#### 2.1.6. Dispersion Stability Analysis

**Figure 3:**
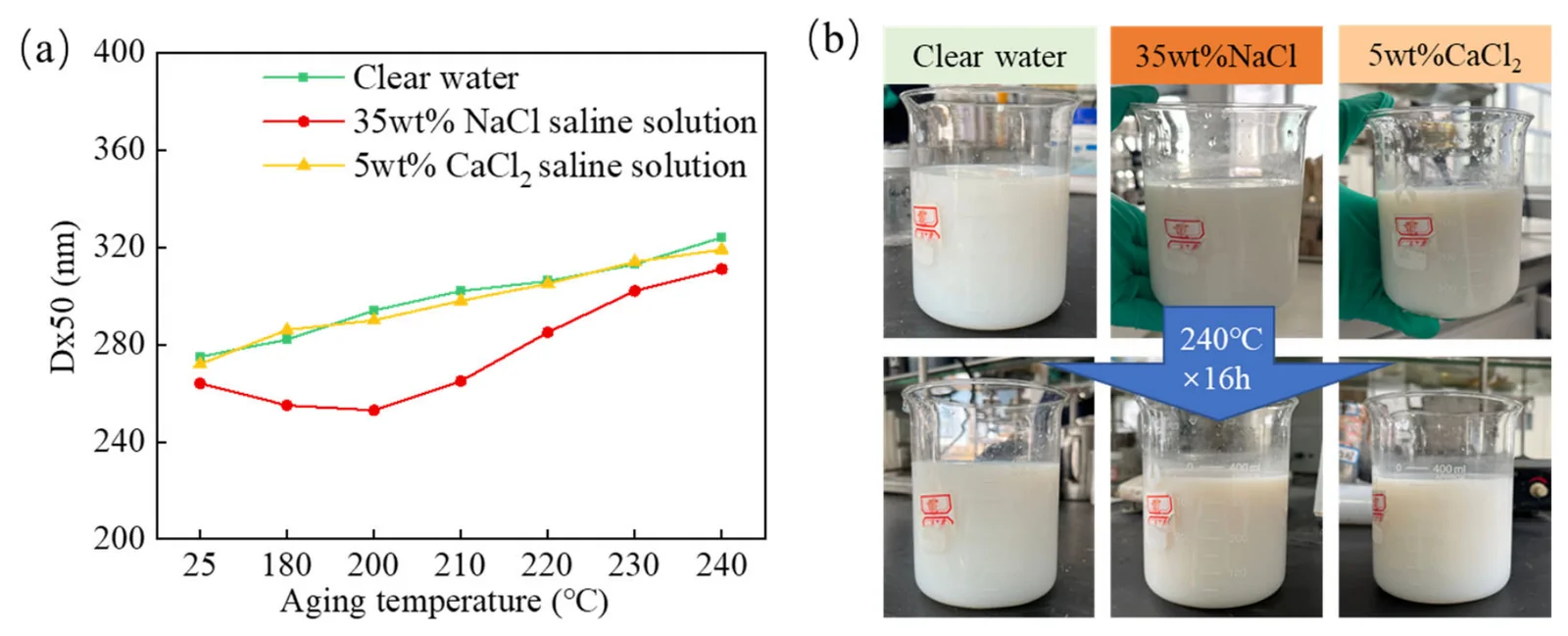
Dispersion stability analysis of HGP: (**a**) Dx50 of HGP clear water suspension and saline suspension at different temperatures, (**b**) Comparison of appearance of suspensions.

In addition to intrinsic structural stability, the dispersion stability of plugging agent particles under high-temperature and high-salinity conditions is another critical factor determining their practical effectiveness [27]. Therefore, the median particle size (Dx50) of 3 wt% HGP dispersions prepared in deionized water, 35 wt% NaCl brine, and 5 wt% CaCl_2_ brine after aging at different temperatures was measured. As shown in Figure 3a, the Dx50 values of HGP dispersions in both freshwater and saline environments remained relatively stable after thermal aging at different temperatures, without significant particle size enlargement. These results indicate that HGP possesses excellent particle size stability under high-temperature and saline conditions. With increasing aging temperature, slight fluctuations in particle size are observed in some systems. This phenomenon is mainly attributed to the enhanced mobility of polymer chains at elevated temperatures, which increases the probability of interparticle collisions [28]. Meanwhile, salt ions can compress the electrical double layer surrounding the particle surfaces, promoting interparticle contact and aggregation. However, the organic–inorganic hybrid structure of HGP effectively suppresses these adverse effects. Specifically, KH570 constructs a stable interfacial connection between the SiO_2_ core and the polymer shell, while the rigid SiO_2_ core provides structural support for the microspheres and restricts excessive deformation of the polymer shell under high-temperature conditions. Furthermore, the sulfonic groups introduced by AMPS and SSS enhance the surface hydration capability and saline-environment adaptability of HGP, enabling the microspheres to maintain stable particle size characteristics under ultra-high temperature and high-salinity conditions. These results are consistent with the intact spherical morphology observed by SEM/TEM after high-temperature aging.

Figure 3b presents the visual appearance of HGP dispersions in deionized water and saline solutions before and after aging at 240 °C for 16 h. It can be observed that the appearance of HGP dispersions remains almost unchanged after high-temperature aging, maintaining a uniform dispersion state without obvious sedimentation, phase separation, or flocculation. These observations directly demonstrate the excellent high-temperature dispersion stability of HGP.

### 2.2. Evaluation of HGP for Improving Drilling Fluid Performance

#### 2.2.1. Influence of HGP on Performance of Basic Fluid

**Figure 4:**
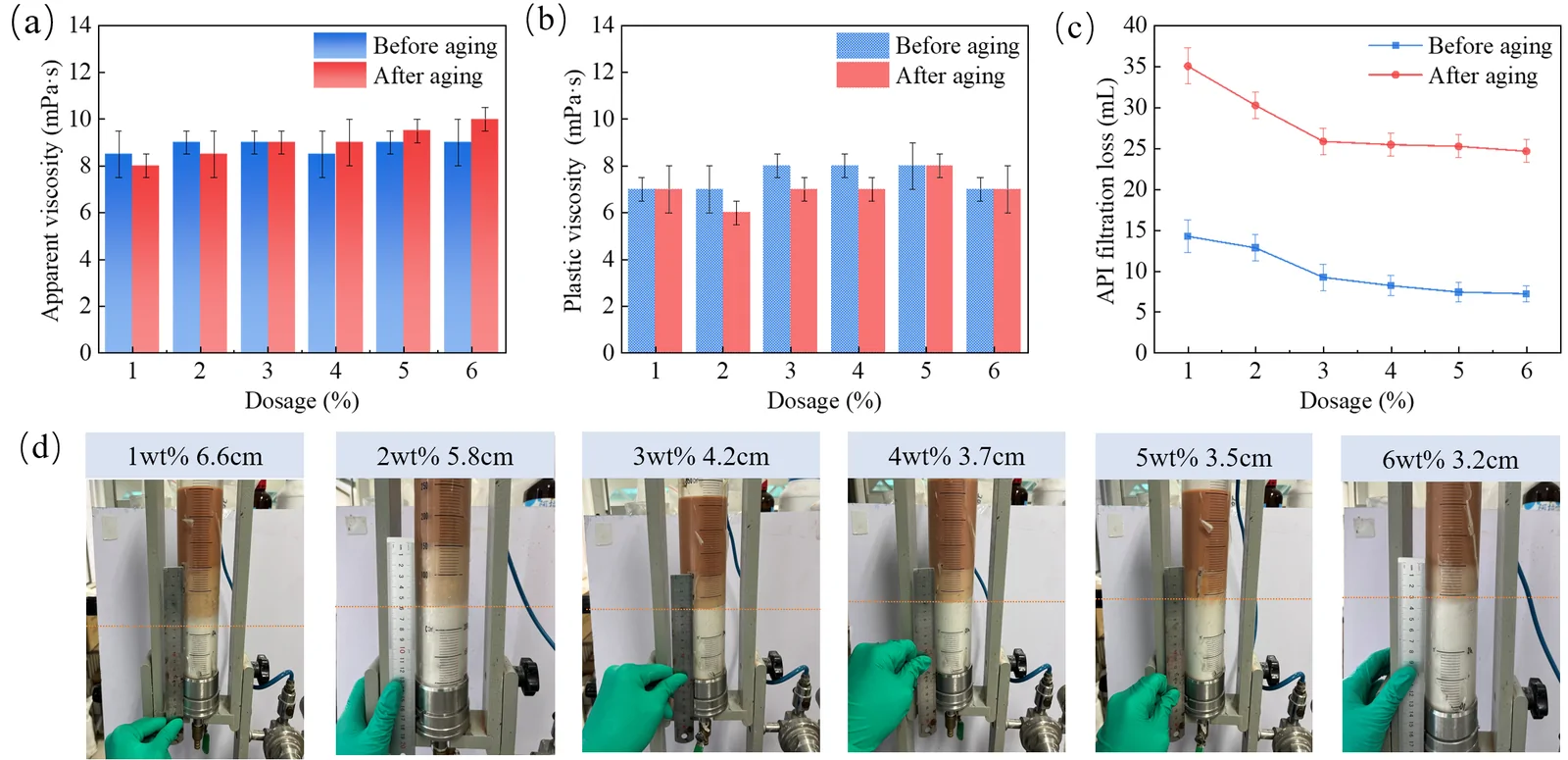
Influence of HGP on performance of basic fluid: (**a**) apparent viscosity, (**b**) plastic viscosity, (**c**) API filtration loss, (**d**) depth of sand bed invasion (test after aging at 240 °C for 16 h).

Figure 4 illustrates the variations in the properties of 4 wt% bentonite-based basic fluids with different HGP concentrations before and after aging at 240 °C. As shown in Figure 4a,b, with increasing HGP concentration from 1 wt% to 6 wt%, the apparent viscosity and plastic viscosity of the drilling fluids exhibit only slight variations. The apparent viscosity gradually increases from approximately 18 mPa·s to 20 mPa·s, while the plastic viscosity remains within the range of 10–12 mPa·s with only minor fluctuations, indicating that the addition of HGP does not cause significant adverse effects on the rheological properties of the drilling fluid. This behavior can be attributed to the core–shell organic–inorganic hybrid structure of HGP. Due to its excellent structural and particle size stability, HGP mainly exists as uniformly dispersed particles in the drilling fluid system and does not significantly interfere with the network framework formed by bentonite particles, thereby avoiding excessive viscosity enhancement. Meanwhile, the hydrophilic polymer chains on the surface of HGP maintain good compatibility with the drilling fluid medium, enabling HGP to improve plugging performance while preserving favorable fluidity of the drilling fluid. This characteristic is particularly important for the field pumping and circulation of ultra-high temperature drilling fluid systems.

As shown in Figure 4c, the API fluid loss of the drilling fluids exhibits a continuous decreasing trend with increasing HGP concentration. When the HGP concentration reaches 3 wt%, the fluid loss decreases by approximately 30%, indicating that a relatively low concentration of HGP can effectively improve the filtration control capability of the drilling fluid after high-temperature aging. The improvement in API fluid loss performance mainly originates from the stable particle size distribution and core–shell structure of HGP. During the filtration process, HGP particles migrate together with bentonite particles toward the filter paper surface and form a denser composite filter cake through interparticle bridging and packing. The resulting compact filter cake reduces permeability and suppresses filtrate invasion. Meanwhile, the flexible polymer shell of HGP can deform and fill the micro-pores within the filter cake, while the SiO_2_ core provides rigid support to enhance the structural stability of the filter cake under high-temperature conditions. Therefore, even after aging at 240 °C, HGP can effectively maintain the filtration control capability of the drilling fluid.

As shown in Figure 4d, the invasion depth of the 240 –aged drilling fluids into the 100–150 mesh sand bed continuously decreases with increasing HGP concentration. When the HGP concentration increases from 1 wt% to 6 wt%, the invasion depth decreases from 6.6 cm to 3.2 cm, corresponding to a reduction of approximately 51.5%. This result demonstrates that HGP can significantly reduce the penetration of drilling fluids into porous media under high-temperature conditions. Specifically, when the HGP concentration increases from 1 wt% to 3 wt%, the invasion depth decreases rapidly from 6.6 cm to 4.2 cm, representing the most significant reduction. This behavior indicates that increasing HGP concentration provides more available particles for rapid pore throat bridging and the formation of an initial plugging layer. With further increasing HGP concentration to 5 wt%, the invasion depth decreases to 3.5 cm, and only slightly decreases to 3.2 cm at 6 wt%, suggesting that effective plugging sites within the sand bed gradually approach saturation, and additional HGP provides limited improvement in plugging efficiency. The excellent sand bed plugging performance of HGP is mainly attributed to its stable particle size distribution and structural integrity under high-temperature conditions. Under differential pressure, HGP particles can enter the pore throats of the sand bed and preferentially form bridging structures at pore entrances. Subsequently, the deformable polymer shell further fills the remaining pore spaces, generating a synergistic “bridging–filling” plugging structure. Meanwhile, the SiO_2_ core enhances the mechanical strength and thermal resistance of the microspheres, preventing particle failure after aging at 240 °C and maintaining the integrity and stability of the plugging layer. Therefore, HGP effectively reduces deep invasion of drilling fluids under high-temperature conditions and contributes to improved wellbore stability.

#### 2.2.2. Evaluation of HGP in Drilling Fluid Under High Temperature and Salt Conditions

**Figure 5:**
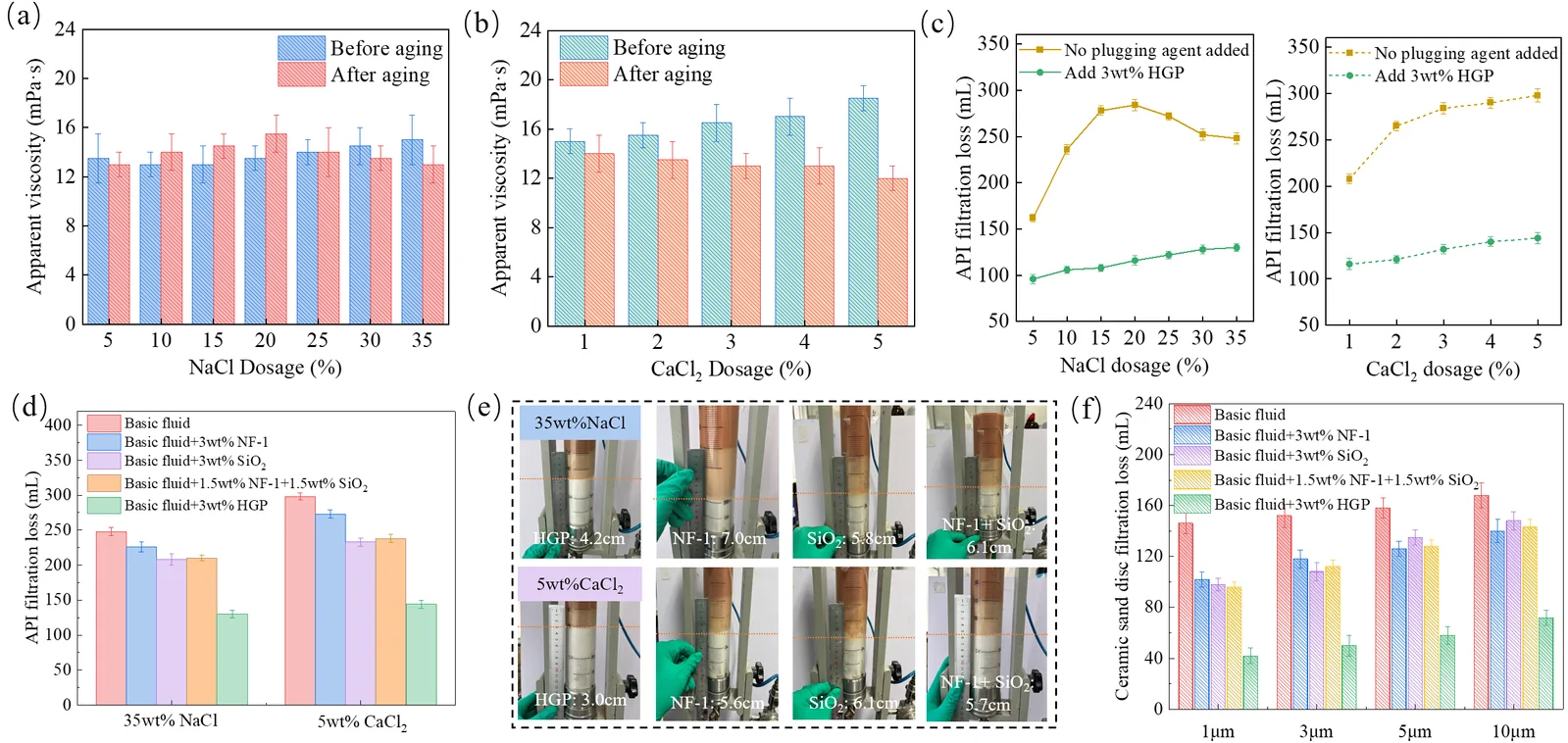
Evaluation of HGP in drilling fluid under high temperature and salt conditions: (**a**) apparent viscosity at different NaCl concentrations, (**b**) apparent viscosity at different CaCl_2_ concentrations, (**c**) API filtration loss at different salt concentrations, (**d**) comparison of API filtration loss, (**e**) comparison of Sand Bed Invasion, (**f**) comparison of filtration loss of ceramic sand discs.

Figure 5a presents the apparent viscosity of the bentonite-based basic fluid containing 3 wt% HGP under different NaCl concentrations. Na^+^ ions can compress the electrical double layer surrounding clay particles, weaken electrostatic repulsion, reduce the hydration capability of bentonite, and promote clay particle aggregation, resulting in significant changes in drilling fluid viscosity. As shown in Figure 5a, the apparent viscosity of the HGP-containing drilling fluid remains relatively stable with increasing NaCl concentration, exhibiting only slight fluctuations. Even at 35 wt% NaCl, no obvious viscosity variation is observed, indicating that well-dispersed HGP particles can effectively alleviate particle aggregation and sedimentation in the drilling fluid under high-temperature and high-salinity conditions.

Figure 5b shows the apparent viscosity variation of the 3 wt% HGP-containing basic fluid under different CaCl_2_ concentrations. Compared with Na^+^, Ca^2+^ possesses stronger ability to compress the electrical double layer and stronger charge interaction capability. It can easily interact with negatively charged sites on bentonite particle surfaces, promoting clay particle aggregation and causing more pronounced changes in drilling fluid rheology. As the CaCl_2_ concentration increases, the apparent viscosity of the HGP-containing drilling fluid exhibits a slight increasing trend, but the overall variation remains relatively moderate. Even under high Ca^2+^ concentrations, the HGP-containing system maintains good rheological stability. This behavior is attributed to the sulfonic groups introduced by AMPS and SSS on the HGP surface, which provide strong hydration ability and maintain surface hydration under high ionic strength conditions. Meanwhile, the rigid SiO_2_ core provides structural support for the polymer shell, reducing polymer chain contraction and structural instability under saline conditions. Therefore, HGP can act as a structural stabilizer within the drilling fluid network framework and partially mitigate irreversible particle aggregation. These results demonstrate that HGP can effectively alleviate the adverse effects of NaCl and CaCl_2_ contamination on drilling fluid properties.

Figure 5c presents the API fluid loss of the HGP-containing basic fluid under different salt concentrations. High concentrations of Na^+^ and Ca^2+^ can deteriorate filter cake quality by reducing clay hydration, weakening electrostatic repulsion, and promoting clay particle aggregation, resulting in larger pore spaces within the deposited filter cake and decreased compactness [29]. As the concentrations of NaCl and CaCl_2_ increase, the API fluid loss gradually increases. However, after adding 3 wt% HGP, the fluid loss decreases significantly, and under highly saline conditions, the HGP-containing drilling fluid exhibits more than 50% reduction in fluid loss compared with the basic fluid. This improvement is attributed to the ability of HGP particles to enter and fill the pores within the filter cake through their suitable particle size and deformability, forming a bridging–filling structure with bentonite particles and reducing filter cake permeability [30]. Meanwhile, the hydrophilic polymer chains on the HGP surface enhance particle interaction and improve filter cake compactness. Therefore, HGP can maintain effective filtration control even under highly mineralized conditions.

To objectively evaluate the plugging performance of HGP, a polymer microsphere plugging agent (NF-1) and an inorganic particle plugging agent (SiO_2_) were selected for comparison. The concentrations of all plugging agents were fixed at 3 wt%. As shown in Figure 5d, under conditions of 35 wt% NaCl and 5 wt% CaCl_2_, HGP exhibits superior fluid loss reduction performance compared with NF-1 and SiO_2_. Although NF-1 possesses a deformable polymer microsphere structure and adaptive plugging capability, its thermal and salt resistance are relatively limited. The absence of an inorganic reinforcing core makes NF-1 more susceptible to polymer chain contraction, dehydration, and structural deformation under high-salinity and high-calcium conditions, resulting in reduced plugging efficiency. In contrast, SiO_2_ particles exhibit excellent thermal stability but are prone to aggregation under high-temperature and high-salinity conditions. Moreover, their rigid structure limits adaptability to heterogeneous pore throat sizes, and weak interparticle interactions restrict their ability to form compact plugging structures. Compared with NF-1 and SiO_2_, HGP integrates the advantages of inorganic rigidity and organic flexibility. The SiO_2_ core ensures structural stability of the microspheres under high-temperature conditions, while the polymer shell provides hydration capability, deformability, and interfacial interaction ability. Therefore, HGP can maintain stable plugging performance under severe saline and calcium-contaminated environments.

The sand bed invasion experiment in Figure 5e further confirms the above analysis. Under the conditions of 35 wt% NaCl and 5 wt% CaCl_2_, the HGP-containing drilling fluid exhibits the lowest sand bed invasion depth, whereas the invasion depths of the NF-1 and SiO_2_ systems are significantly higher, demonstrating the superior plugging capability of HGP. This result indicates that HGP can not only reduce filtration loss but also effectively inhibit the penetration of drilling fluids into porous media. This behavior is attributed to the ability of HGP microspheres to enter pore throats and achieve pore filling through elastic deformation. Meanwhile, multiple HGP particles can form stable bridging structures, and the SiO_2_ core enhances the compressive resistance of microspheres and reinforces the plugging layer. In comparison, NF-1 mainly relies on polymer elasticity for plugging, resulting in insufficient structural stability under saline and calcium-containing environments. Although SiO_2_ particles possess high rigidity, their limited deformability restricts their ability to form compact plugging structures.

To further evaluate the adaptability of HGP-containing drilling fluids to pore throat structures with different sizes, ceramic sand discs with different pore sizes were employed to simulate formations with different permeability characteristics. The filtration loss of drilling fluids containing 3 wt% HGP, NF-1, and SiO_2_ was measured, and the results are presented in Figure 5f. With changes in ceramic sand disc pore size, the filtration loss of all three plugging agent systems exhibits different degrees of variation. When the sand disc pore size decreases, the corresponding pore throat size becomes smaller, allowing plugging particles to more easily bridge the pore entrances and form compact external plugging layers together with bentonite particles, thereby reducing further filtrate invasion. In contrast, under larger pore size conditions, the increased pore space makes it more difficult for plugging particles to rapidly establish effective bridging structures or causes more particles to migrate with the filtrate, resulting in relatively higher filtration loss.

Compared with the basic fluid and drilling fluids containing NF-1 or SiO_2_, the HGP-containing system consistently maintains lower filtration loss under different sand disc conditions. The filtration loss of the HGP system is less than 50% of that of NF-1 and SiO_2_ systems, indicating its superior plugging performance. The differences among the three plugging agents further reflect their distinct structural characteristics. For NF-1 polymer microspheres, the filtration loss gradually becomes lower than that of the SiO_2_ system with increasing sand disc pore size, indicating that NF-1 possesses certain deformable filling capability. The polymer chains can be compressed and adapt to pore structures, thereby improving plugging efficiency. However, because NF-1 mainly relies on the polymer network to provide plugging strength, its performance can be affected by polymer chain contraction and structural instability under high-temperature and high-salinity conditions [31], resulting in inferior overall plugging performance compared with HGP. SiO_2_ particles exhibit excellent thermal stability, but their plugging mechanism mainly depends on particle size matching and mechanical accumulation. In smaller pore size sand discs, SiO_2_ particles can more easily accumulate at pore entrances and form compact plugging structures, resulting in significantly reduced filtration loss. However, due to their rigid structure and lack of deformability, the interactions between particles are relatively weak, making it difficult to maintain stable plugging structures in larger pore spaces [32]. Consequently, their plugging performance is inferior to that of HGP. In contrast, HGP exhibits superior plugging performance across ceramic sand discs with different pore sizes. This advantage originates from the synergistic effect of its organic–inorganic core–shell structure. The SiO_2_ core provides rigid support and enhances the structural stability of microspheres under high-temperature and high-pressure conditions, while the polymer shell endows HGP with elastic deformation capability, enabling adaptation to different pore throat sizes and close contact with sand particles. In small-pore sand discs, HGP can rapidly establish bridging structures and simultaneously utilize the deformable polymer shell to fill interparticle gaps, forming a composite plugging layer characterized by “particle bridging–elastic filling–compaction stabilization”. As a result, HGP achieves the lowest filtration loss among the tested plugging agents.

To further verify the synergistic effect arising from the organic–inorganic core–shell structure of HGP and exclude the possibility that its plugging performance is merely derived from the simple combination of polymeric microspheres and inorganic SiO_2_ particles, a control group containing 1.5 wt% NF-1 and 1.5 wt% SiO_2_ was prepared, with the total dosage of the two plugging agents kept equal to that of 3 wt% HGP. As shown in Figure 5d–f, under 35 wt% NaCl and 5 wt% CaCl_2_ contamination conditions, the API fluid loss, sand bed invasion depth, and ceramic sand disc filtration loss of the combined control group were generally intermediate between those of the NF-1 and SiO_2_ groups, whereas the HGP group consistently exhibited superior performance. In the API fluid loss test, although the combined group showed lower fluid loss than the NF-1 group, its fluid loss remained considerably higher than that of the HGP group. In the sand bed plugging test, the invasion depth of the combined group was likewise between those of the NF-1 and SiO_2_ groups, while the HGP group exhibited the lowest invasion depth. For ceramic sand discs with different pore sizes, the filtration loss of the combined group remained within the corresponding ranges of the NF-1 and SiO_2_ groups, whereas the HGP group consistently maintained the lowest filtration loss, indicating its superior adaptability to and plugging capability for pore throats of different sizes. These results demonstrate that, at the same total dosage, the simple combination of NF-1 and SiO_2_ does not produce plugging performance comparable to that of HGP, indicating that the enhanced performance of HGP is not simply attributable to the physical combination of the two plugging components, but rather to the synergistic effect arising from its organic–inorganic core–shell structure.

#### 2.2.3. Microscopic Influence Mechanism of HGP on Drilling Fluid

To elucidate the microscopic mechanism by which HGP regulates the plugging performance of drilling fluids under high-temperature and high-salinity conditions, particle size analysis, zeta potential measurement, filter cake permeability evaluation, and microscopic morphology observation were performed for HGP-containing drilling fluids under different environmental conditions.

**Figure 6:**
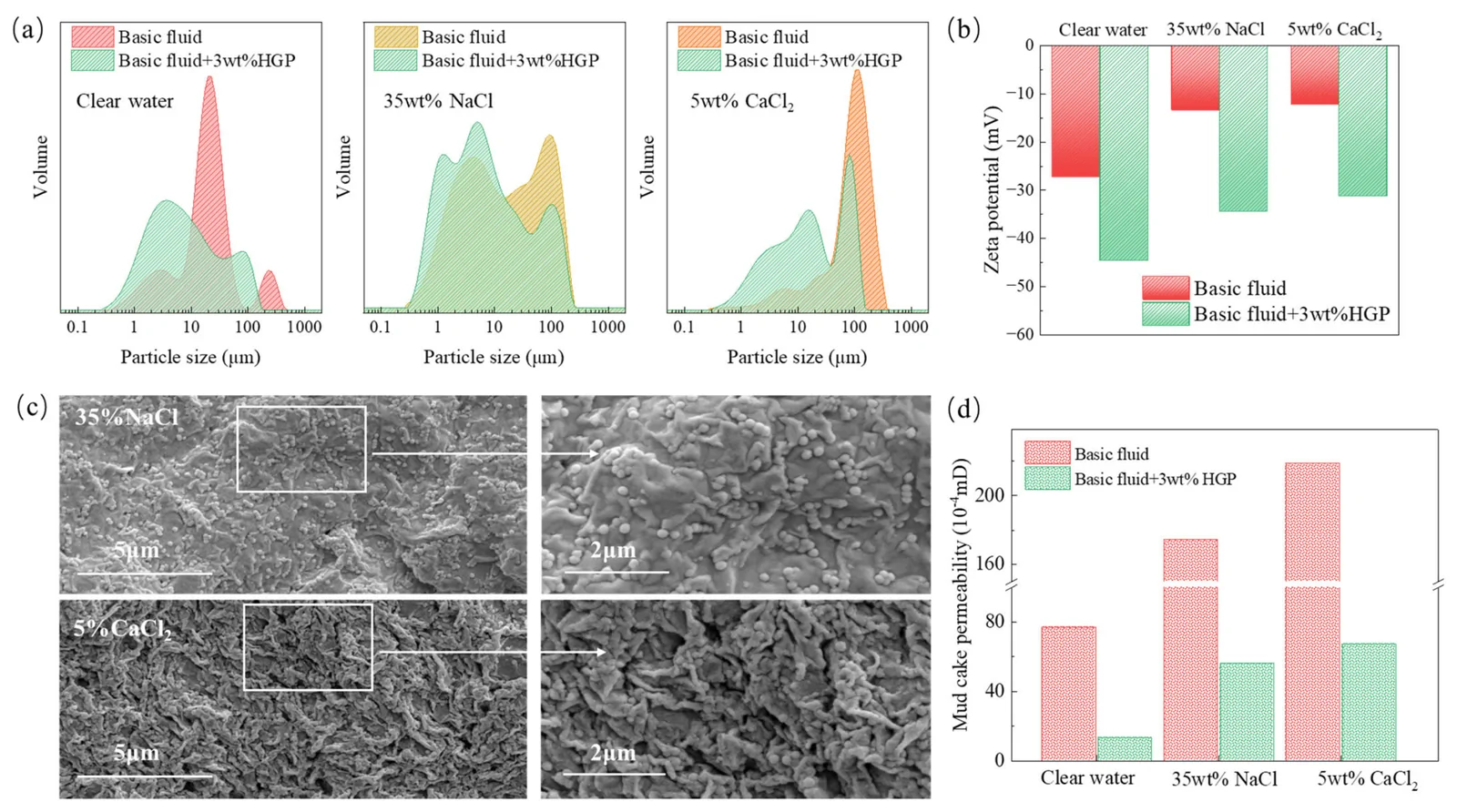
Microscopic influence mechanism of HGP on drilling fluid: (**a**) particle size analysis, (**b**) Zeta potential analysis, (**c**) microscopic morphology analysis of mud cake, (**d**) analysis of mud cake permeability.

Figure 6a presents the effect of HGP on the particle size distribution of drilling fluids after aging at 240 °C under different saline environments. Under high-temperature and high-salinity conditions, the diffuse double layer surrounding clay particles is compressed, weakening the electrostatic repulsion between particles. As a result, bentonite particles tend to aggregate and form larger flocculated structures, leading to a shift of particle size distribution toward larger dimensions. As shown in Figure 6a, HGP effectively regulates the particle size distribution of aged drilling fluids in deionized water, 35 wt% NaCl brine, and 5 wt% CaCl_2_ brine. Compared with the basic fluid without HGP, the HGP-containing systems exhibit a more uniform particle size distribution and a reduced proportion of large aggregated particles. This behavior can be attributed to two aspects. First, the hydrophilic polymer chains on the HGP surface can adsorb onto clay particle surfaces and inhibit particle aggregation through steric hindrance. Second, HGP itself possesses a stable nano/submicron-scale particle structure and can participate in the regulation of solid particle size distribution as a plugging component, enabling a more optimized particle packing arrangement in the drilling fluid system and providing favorable particle foundations for the formation of compact filter cakes [33].

Figure 6b shows the zeta potential results of the corresponding drilling fluids. Zeta potential reflects the electrostatic repulsion between particles. Under high-temperature and high-salinity conditions, a lower absolute zeta potential value indicates weakened electrostatic repulsion, which promotes particle aggregation and deterioration of filter cake structure. Compared with the basic fluid, the addition of HGP significantly increases the absolute zeta potential value of aged drilling fluids, indicating that HGP enhances the surface charge stability of particles within the system. The strongly hydrophilic anionic groups on HGP can be exposed on particle surfaces, increasing the surface negative charge density and strengthening electrostatic repulsion between particles. Meanwhile, the polymer shell of HGP can form a hydrated protective layer around particles, further improving particle stability under high ionic strength conditions. Therefore, HGP effectively enhances the dispersion stability of solid particles in drilling fluids, resulting in higher absolute zeta potential values [34].

Scanning electron microscopy was further employed to observe the microscopic morphology of filter cakes obtained from drilling fluids aged at 240 °C under 35 wt% NaCl and 5 wt% CaCl_2_ conditions. Under high-temperature and high-salinity environments, the hydration capability of clay particles decreases, leading to loosely arranged particles and the formation of interconnected filtration channels within the filter cake. As shown in Figure 6c, HGP particles remain tightly attached to the filter cake surface under saline conditions and effectively fill the pores and cracks within the filter cake. By blocking filtration pathways, HGP promotes the formation of a more uniform, compact, and stable composite filter cake, directly revealing its microscopic mechanism for improving filtration control. To quantitatively evaluate the effect of HGP on filter cake compactness under high-temperature and high-salinity conditions, the permeability of filter cakes obtained from the basic fluid and the 3 wt% HGP-containing fluid after aging at 240 °C was measured. As shown in Figure 6d, the addition of HGP significantly decreases filter cake permeability under different salt and calcium contamination conditions, with reductions exceeding 70%. This result further confirms that HGP can effectively seal internal filtration channels within filter cakes under extreme conditions, thereby reducing fluid seepage pathways.

Based on the above analysis, the microscopic mechanism of HGP in regulating drilling fluid plugging performance under ultra-high temperature and high-salinity conditions can be summarized into three aspects: stabilization of particle dispersion, enhancement of surface charge stability, and optimization of filter cake microstructure. The sulfonic groups on HGP improve the dispersion stability of particles in drilling fluids, while the polymer shell enhances filter cake structure through steric stabilization and flexible filling effects. Meanwhile, the SiO_2_ core provides structural support under high-temperature conditions. The synergistic contribution of these components enables HGP to construct a compact, low-permeability, and highly stable composite filter cake, thereby enhancing the plugging capability of ultra-high temperature and high-salinity water-based drilling fluids.

### 2.3. HGP Plugging Ability and Mechanism Evaluation

#### 2.3.1. Microporous Membrane Plugging Analysis

Water-based drilling fluids are complex colloidal systems containing clay particles, weighting materials, and various treatment agents [35]. During thermal aging, the macroscopic properties of drilling fluids (e.g., rheological properties and filtration behavior) are affected by multiple coupled factors, including particle aggregation, additive degradation, and interactions among different components [36]. Therefore, changes in drilling fluid performance alone may not fully reflect the intrinsic plugging ability of individual plugging agents. To further investigate the intrinsic plugging capability and plugging mechanism of HGP, the influence of other drilling fluid components was eliminated, and the plugging behavior of HGP particles alone was directly evaluated. A 3 wt% aqueous dispersion of HGP, NF-1, and SiO_2_ was prepared, respectively, and aged at 240 °C for 16 h. The filter paper used in the API filtration test was replaced with PTFE microporous membranes with different pore sizes to evaluate the filtration behavior of different plugging agent dispersions.

**Figure 7:**
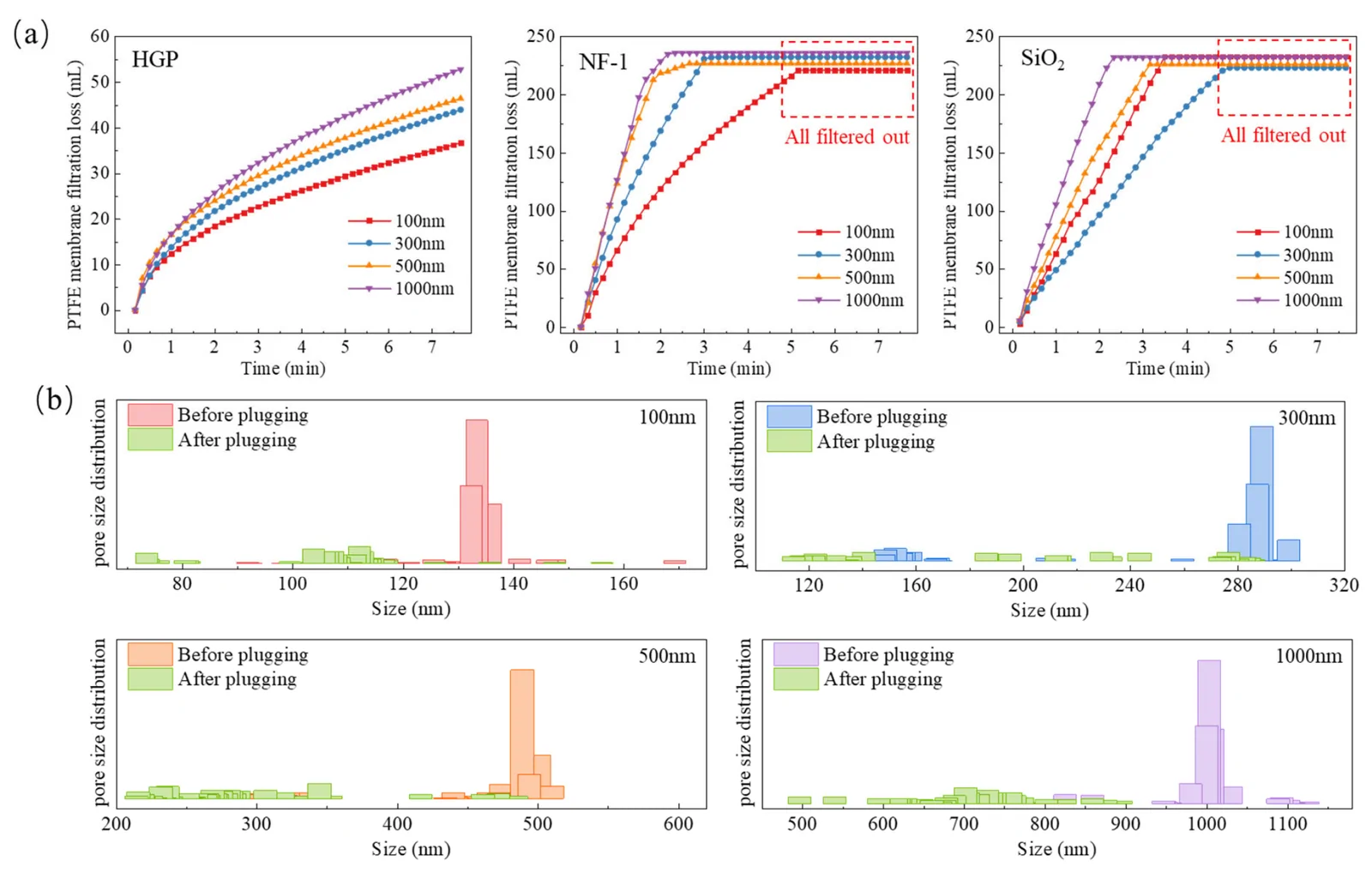
Microporous membrane plugging analysis: (**a**) PTFE membrane filtration loss, (**b**) membrane pore size analysis.

Figure 7a presents the variation of filtration volume with time for the dispersions passing through PTFE microporous membranes with pore sizes of 100, 300, 500, and 1000 nm. Significant differences were observed among the three plugging agents. For the HGP dispersion, the filtration rate decreased rapidly for all membrane pore sizes and reached a stable state within a relatively short period, indicating that HGP particles can effectively adapt to different pore throat sizes and form stable plugging structures on the membrane surface. In contrast, the NF-1 and SiO_2_ dispersions exhibited insufficient initial bridging efficiency and poor integrity of the formed plugging layers, resulting in continuous leakage of the suspensions over time. As a polymer microsphere plugging agent, NF-1 possesses certain elastic deformation capability and can enter pore channels through compression deformation, thereby achieving pore filling and providing a certain plugging effect for membranes with different pore sizes [37]. However, its plugging process mainly relies on the polymer network structure. Under high-temperature conditions and without rigid structural support, the compressive resistance and structural stability of NF-1 particles are limited, making it difficult to rapidly construct a stable plugging layer [38]. The SiO_2_ particles achieve plugging mainly through mechanical bridging between rigid particles. When the particle size is well matched with the membrane pore size, effective bridging structures can be formed. However, due to the lack of deformability and weak interparticle interactions, SiO_2_ particles tend to form loosely packed structures with interconnected pores, resulting in limited filtration control capability that strongly depends on pore size matching [39]. In comparison, HGP combines the advantages of both organic and inorganic components. The SiO_2_ core provides rigid structural support, allowing the microspheres to maintain their integrity under pressure, while the polymer shell provides flexibility and enables moderate deformation and pore filling. Consequently, HGP can construct denser and more stable plugging layers under different pore size conditions.

The pore size distributions of PTFE membranes before and after HGP plugging were further analyzed. As shown in Figure 7b, the untreated PTFE membranes exhibit relatively concentrated initial pore size distributions. After treatment with HGP dispersion, the pore size distribution of the membranes shifts significantly toward smaller dimensions, accompanied by the disappearance of some larger pore size peaks, indicating that HGP particles effectively enter and fill membrane pores, resulting in pore size reduction. This change demonstrates that HGP does not simply form a surface coating layer on the membrane but actively regulates the original pore structure through particle invasion, pore entrance bridging, and subsequent particle accumulation. During the plugging process, HGP particles initially establish bridging structures at pore entrances. Subsequently, deformation of the polymer shell further fills the micropores within the bridging framework, while the rigid SiO_2_ core provides continuous structural support. With progressive compaction, a dense composite plugging layer with low permeability is ultimately formed.

#### 2.3.2. Core Plugging Analysis

**Figure 8:**
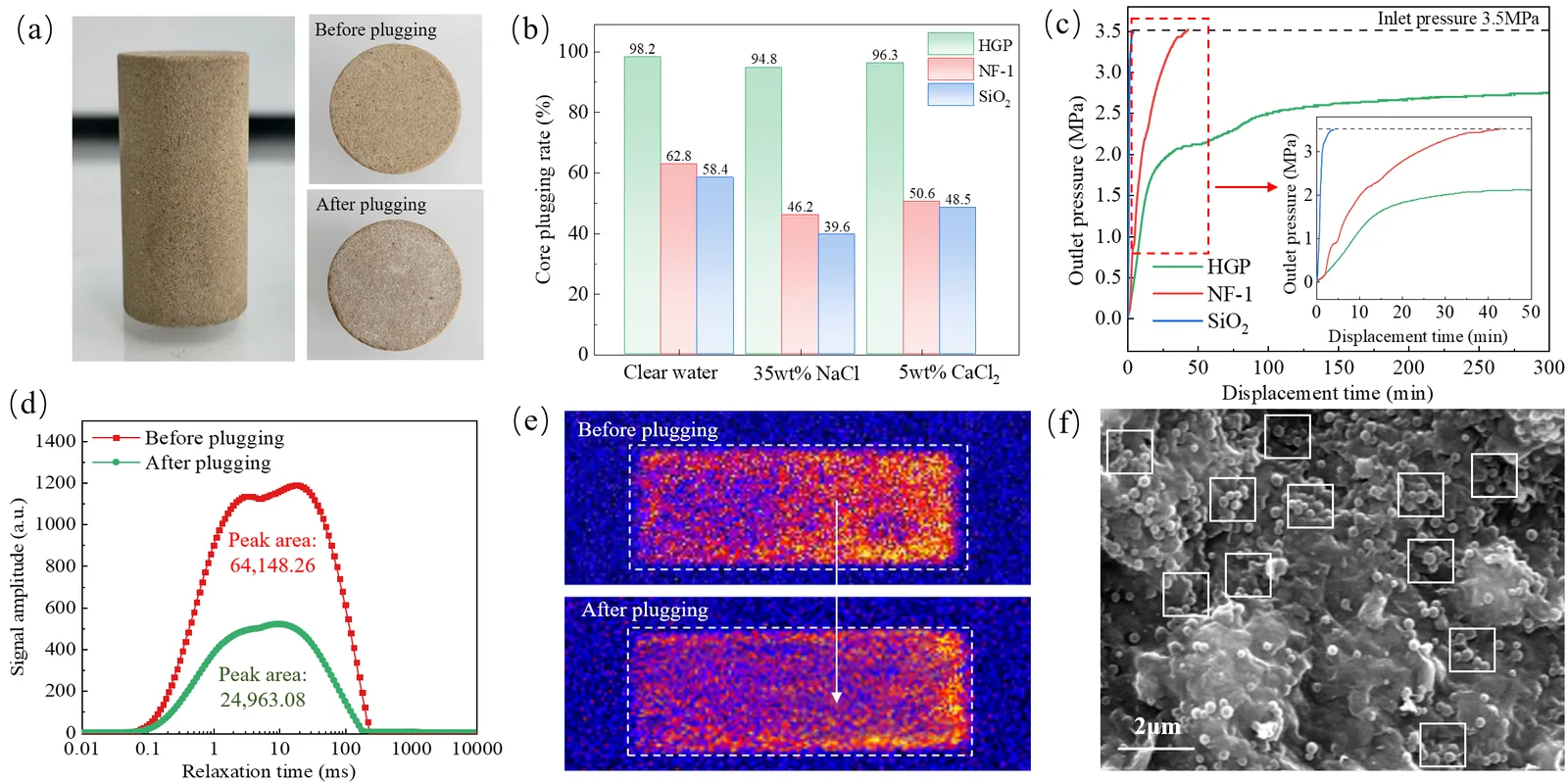
Core plugging analysis: (**a**) artificial sandstone core used, (**b**) core plugging rate, (**c**) high temperature in-situ pressure transmission, (**d**) core nuclear magnetic T2 spectrum, (**e**) core magnetic resonance imaging, (**f**) SEM image of internal cross-section of core.

In actual drilling operations, the primary targets of plugging agents are pores and microfractures within wellbore formations. To further evaluate the plugging capability of HGP toward geological porous media, artificial sandstone cores with an average permeability of 10 mD, as shown in Figure 8a, were selected for core plugging evaluation.

Figure 8b presents the core plugging efficiencies of HGP, NF-1, and SiO_2_ dispersions prepared in deionized water, 35 wt% NaCl brine, and 5 wt% CaCl_2_ brine after aging at 240 °C for 16 h. It can be observed that HGP exhibits core plugging efficiencies above 94% under all tested conditions, which are significantly higher than those achieved by NF-1 and SiO_2_. These results demonstrate that HGP maintains excellent plugging performance under both freshwater and highly saline/calcium-containing environments.

Among these conditions, the 35 wt% NaCl dispersion exhibited the lowest plugging efficiency for HGP and was therefore selected for further in situ pressure transmission tests at 240 °C. The pressure transmission test evaluates fluid migration resistance within porous media by monitoring the response rate of internal core pressure under external pressure loading. A longer time required for the inlet and outlet pressures to reach equilibrium indicates greater resistance along the fluid migration pathway or fewer available transport channels within the core [40]. As shown in Figure 8c, the outlet pressure of the HGP-plugged core increases at the slowest rate, and the inlet and outlet pressures remain unbalanced even after 300 min. The pressure transmission experiment was continuously conducted for approximately 50 h under 240 °C conditions. In contrast, the NF-1 and SiO_2_ systems reach pressure equilibrium within 40 min. This result indicates that HGP forms a denser and more stable plugging structure inside the core, effectively delaying pressure propagation and restricting fluid migration.

Nuclear magnetic resonance (NMR) analysis was simultaneously performed on the HGP-treated core before and after the pressure transmission test. NMR hydrogen signals can be used to determine water saturation within porous media. The transverse relaxation time (T_2_) is proportional to pore size, while the signal amplitude corresponds to the amount of fluid contained within pores of specific sizes [41]. The integrated peak area of the T_2_ spectrum is proportional to the total volume of fluid-accessible pores within the core. Therefore, variations in T_2_ distributions can reveal changes in fluid distribution among pores with different sizes, while changes in peak area can reflect variations in effective pore volume after plugging [15]. As shown in Figure 8d, the T_2_ spectra of the water-saturated core before and after HGP plugging exhibit significant differences. After plugging, the overall signal intensity decreases substantially, and the integrated peak area decreases from 64,148.26 to 24,963.08, corresponding to a reduction of more than 60%. This indicates that a large proportion of the pore space inside the core is occupied by plugging materials or becomes disconnected after HGP treatment, resulting in a significant decrease in fluid-accessible pore volume and confirming effective internal plugging. NMR imaging was further employed to visualize the spatial distribution of water within the core. Through pseudo-color processing, different colors represent different water saturation states, where red/orange regions correspond to water-saturated areas and blue regions represent regions with little or no water. As shown in Figure 8e, the red/orange regions within the core are significantly reduced after HGP treatment, directly demonstrating that HGP achieves deep and stable plugging inside the core by blocking connected pore pathways and preventing water invasion.

After the pressure transmission test, the HGP-treated core was fractured and the internal structure was examined by scanning electron microscopy. As shown in Figure 8f, HGP particles can be clearly observed distributed within the pore spaces of the sandstone matrix. Further observation of the particle aggregation region highlighted by the white rectangle reveals that HGP particles form bridging structures and accumulate with each other, filling pore spaces and constructing relatively continuous plugging networks. These observations directly confirm the ability of HGP to establish effective internal plugging structures within porous media.

#### 2.3.3. Summary of HGP Action Mechanism

**Figure 9:**
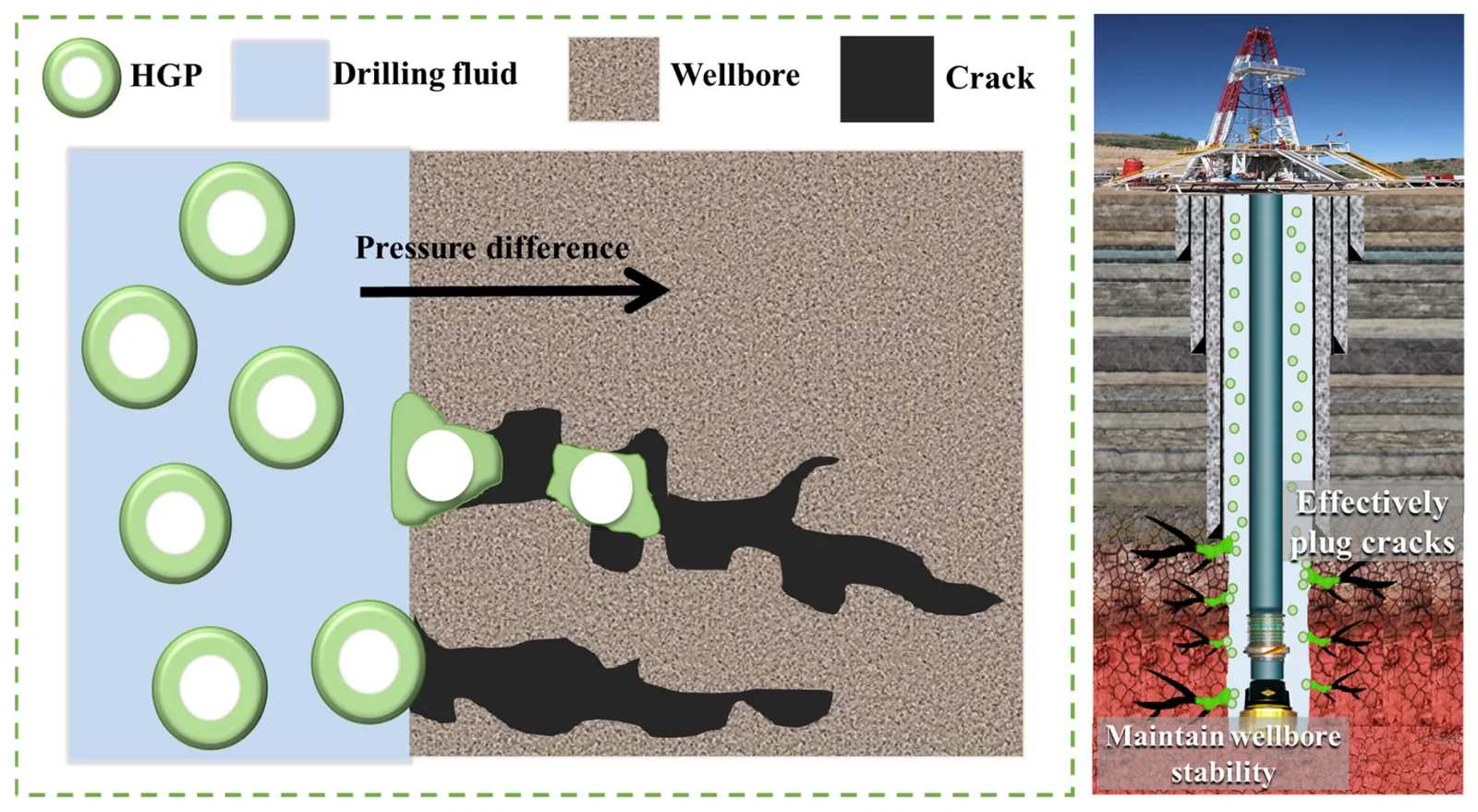
Schematic diagram of HGP.

As shown in Figure 9, HGP possesses an organic–inorganic core–shell structure consisting of a flexible polymer shell and a rigid SiO_2_ core, enabling efficient plugging under ultra-high temperature and high-salinity conditions through a synergistic mechanism involving dispersion stabilization, pore-throat bridging, deformation filling, and structural stabilization. The unique chemical composition and hierarchical structure of HGP provide excellent thermal stability and mechanical strength. The hydrophilic sulfonic groups within the polymer shell enhance particle hydration and surface charge stability, allowing HGP to maintain stable particle size distribution and good dispersion under 240 °C, high-salinity, and high-calcium conditions, thereby preventing particle aggregation and sedimentation. Driven by pressure differentials, HGP particles migrate with drilling fluids into formation pores and microfractures, where they rapidly form bridging structures at pore throats. Subsequently, the flexible polymer shell undergoes deformation and fills the remaining void spaces, while the rigid SiO_2_ core provides mechanical support to stabilize the plugging framework. Meanwhile, within the drilling fluid system, HGP optimizes the solid particle size distribution and improves particle dispersion stability, promoting the formation of a continuous, compact, and low-permeability filter cake, thereby further reducing filtrate invasion. Consequently, the HGP-induced plugging structure integrates high mechanical strength with adaptive deformation capability, effectively blocking fluid migration through pores and microfractures and maintaining wellbore stability under ultra-high temperature and high-salinity drilling conditions.

## 3. Conclusions

1. An organic–inorganic hybrid gel microsphere plugging agent HGP is successfully prepared via emulsion polymerization using KH570-modified nano-SiO_2_ as the inorganic component and AMPS, St, and SSS as organic monomers. The characterization results from FTIR, XPS, ^1^H NMR, TG, SEM, and TEM demonstrate that HGP possesses the designed organic–inorganic hybrid core–shell structure, consisting of a nano-SiO_2_ inorganic core and a flexible copolymer gel shell. The SiO_2_ core provides excellent rigid support and structural stability for the microspheres, while the benzene ring structures in the polymer shell enhance the rigidity of polymer chains and thermal stability. Meanwhile, the sulfonic acid groups introduced by AMPS and SSS endow HGP with excellent hydrophilicity and salt resistance. The synergistic effects of these structural components enable HGP to maintain stable structural integrity under ultra-high-temperature and high-salinity conditions.
2. HGP effectively improves the plugging performance of ultra-high-temperature and high-salinity water-based drilling fluids while maintaining rheological stability. Under 240 °C thermal aging and high-concentration NaCl and CaCl_2_ contamination conditions, HGP maintains a stable particle size distribution and excellent dispersion stability, effectively inhibiting salt ion-induced particle aggregation. The addition of HGP has little influence on the apparent viscosity and plastic viscosity of drilling fluids but significantly reduces fluid loss and improves the compactness and plugging performance of filter cakes. This improvement is mainly attributed to the optimization of solid particle size distribution by HGP and its capability to promote the formation of a dense and low-permeability structure during filter cake construction.
3. HGP exhibits excellent plugging performance for pores and fractures with different scales and types, and its comprehensive performance is superior to that of the conventional polymer microsphere plugging agent NF-1 and inorganic SiO_2_ particles. Benefiting from its stable particle size distribution and rigid–flexible integrated core–shell structure, HGP can enter pore throats with different sizes under pressure and achieve adaptive plugging through particle bridging and deformation filling. Compared with NF-1 and SiO_2_, HGP exhibits higher plugging efficiency, environmental adaptability, and structural stability under high-temperature and high-salinity conditions, enabling the formation of a more complete, compact, and stable plugging barrier.
4. The enhanced performance was considered to arise from the synergistic effects of stable dispersion, pore-throat bridging, deformation filling, and structural stabilization. Driven by pressure differences, HGP can enter formation pores and microfractures along with drilling fluids and form bridging structures at pore throats. Subsequently, the flexible deformation of the polymer shell enables the accumulation and filling of pore spaces, while the rigid SiO_2_ core provides stable mechanical support, constructing a dense plugging layer and improving the stability of the plugging structure under ultra-high-temperature and high-pressure conditions. This rigid–flexible synergistic structural design strategy provides a new approach for the development of high-performance plugging materials applicable to extreme geological conditions.

## 4. Materials and Methods

### 4.1. Materials

Styrene (St, AR), 2-acrylamido-2-methyl-1-propanesulfonic acid (AMPS, AR), sodium p-styrenesulfonate (SSS, AR), and ammonium persulfate (APS, AR) were purchased from Shanghai Aladdin Biochemical Technology Co., Ltd., Shanghai, China. KH570-modified nano-SiO_2_ (KH570-SiO_2_) was purchased from Jiangsu XFNANO Materials Tech Co., Ltd., Nanjing, China. Sodium carbonate (Na_2_CO_3_, AR), sodium hydroxide (NaOH, AR), sodium chloride (NaCl, AR), and calcium chloride (CaCl_2_, AR) were purchased from Sinopharm Chemical Reagent Co., Ltd., Shanghai, China. Drilling fluid-grade premium bentonite and nano-SiO_2_ plugging agent (Near spherical, median particle size 250nm) for drilling fluids were purchased from Chongqing Weining Drilling Additives Co., Ltd., Chongqing, China. [18]. The high-temperature-resistant polymer microsphere plugging agent NF-1 (Near spherical polymer microsphere lotion, microsphere content is about 35%, median particle size 260nm) was purchased from Beijing Kenuojie Energy Environmental Protection Technology Co., Ltd., Beijing, China. [13]. Artificial cores, ceramic sand discs, filter papers, and microporous membranes used for performance evaluation were purchased from Qingdao Xusheng Petroleum Instrument Co., Ltd., Qingdao, China.

### 4.2. Structural Design of Organic Inorganic Hybrid Gel Microsphere

To overcome the inherent limitations of conventional polymer microsphere plugging agents and inorganic particle plugging agents in ultra-high-temperature and high-salinity drilling fluids, an organic–inorganic hybrid plugging agent design strategy was proposed in this study, and an organic–inorganic hybrid gel microsphere HGP with a core–shell structure was successfully constructed. KH570-modified nano-SiO_2_ was used as the inorganic core, and a polymer gel shell was constructed in situ on the surface of SiO_2_ through the copolymerization of the methacryloxy group in KH570 with AMPS, St, and SSS, achieving a robust integration between the organic and inorganic phases. In this structural design, the SiO_2_ core acts as an inorganic reinforcing phase, providing the microspheres with excellent thermal stability, mechanical strength, and structural support, thereby maintaining their structural integrity under ultra-high-temperature and high-pressure conditions. The rigid benzene rings introduced by St and SSS enhance the rigidity and thermal resistance of the polymer backbone, reduce the mobility of polymer chains at elevated temperatures, and improve the thermal stability of the polymer framework. The abundant sulfonic acid groups introduced by AMPS and SSS possess strong hydration ability and salt tolerance, which can effectively mitigate ion shielding effects under high-salinity conditions, maintain the dispersion stability of the microspheres, and enhance their interaction with solid particles in drilling fluids. Meanwhile, the polymer shell endows the microspheres with certain flexibility and deformability, enabling them to adapt to pore throats with different sizes and achieve synergistic bridging and filling during the plugging process. The SiO_2_ core is considered to contribute to the structural stability of the plugging layer by providing rigid support under high-temperature and high-pressure conditions. Through this structural design, HGP integrates the high strength and excellent thermal resistance of inorganic particles with the flexible deformation and interfacial adaptability of polymer microspheres. As a result, HGP can maintain stable dispersion and form a dense and stable plugging layer under ultra-high-temperature and high-salinity conditions, providing a new organic–inorganic synergistic reinforcement strategy for the design of high-performance plugging agents for water-based drilling fluids under extreme conditions.

### 4.3. Preparation of HGP

**Figure 10:**
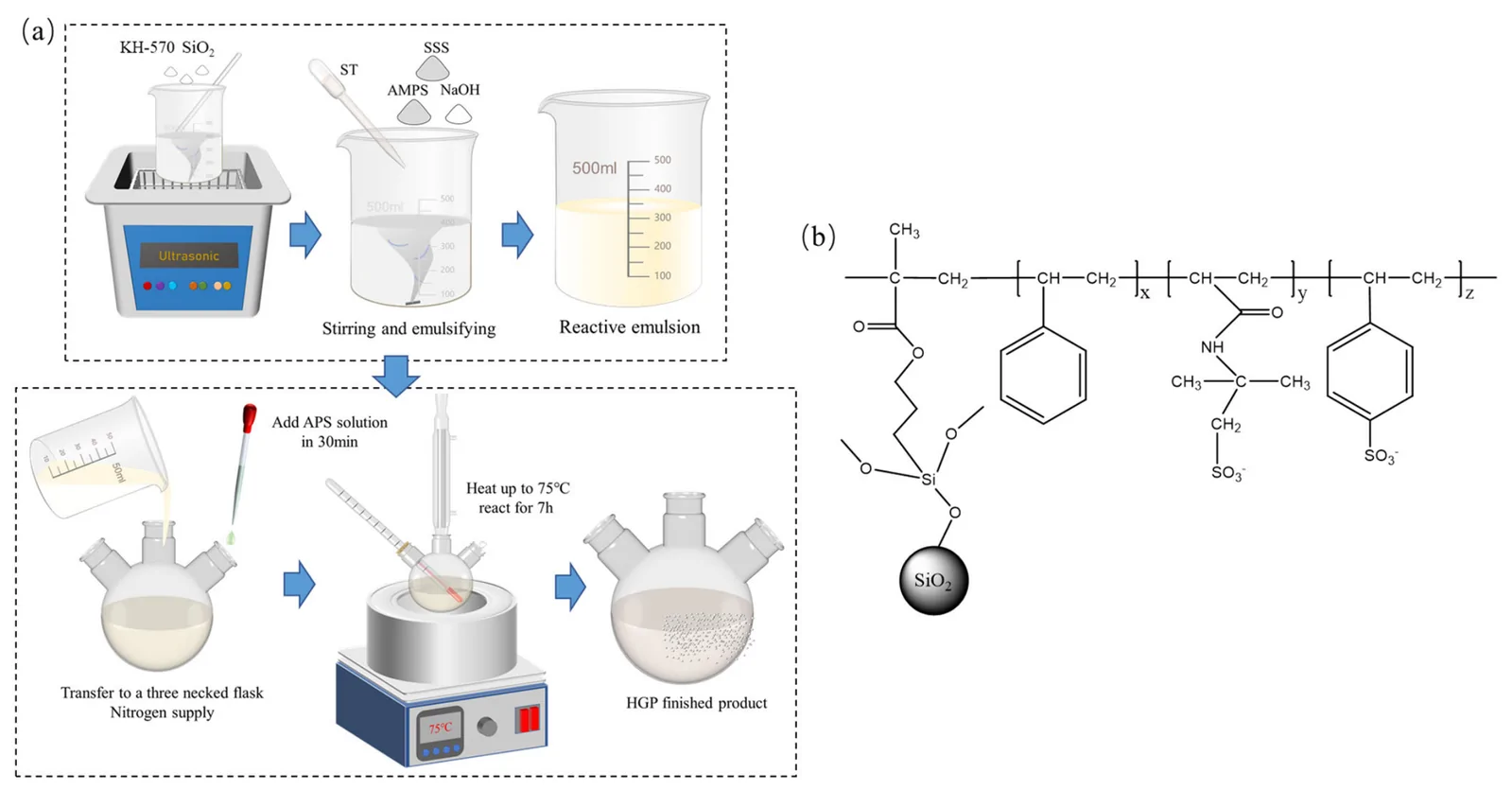
Preparation of HGP: (**a**) preparation flowchart, (**b**) schematic diagram of HGP molecular structure.

HGP was synthesized via a soap-free emulsion polymerization method, and the detailed synthesis procedure is described as follows. First, KH570-modified nano-SiO_2_ (0.5 wt% relative to deionized water) was added into deionized water and ultrasonically dispersed under stirring for 10 min. Subsequently, AMPS (8 wt% relative to deionized water) was added and continuously stirred until complete dissolution, followed by adjusting the pH of the solution to 7–8 using NaOH. Then, SSS (2 wt% relative to deionized water) was added and dissolved completely, after which St (20 wt% relative to deionized water) was introduced. The stirring speed was increased to approximately 500 rpm, and the mixture was emulsified for 20 min. The obtained emulsion was transferred into a three-neck flask, followed by nitrogen purging to remove dissolved oxygen. The flask was placed in a water bath and heated under continuous stirring. When the temperature reached 75 °C, an APS solution (0.5 wt% relative to deionized water, with a concentration of 50%) was added to initiate polymerization. The reaction was maintained at 75 °C under a nitrogen atmosphere with continuous stirring for 7 h to obtain the final HGP dispersion emulsion (The effective content of HGP microspheres is about 35%). During practical application, the HGP dispersion emulsion was directly added into drilling fluids according to its mass. The synthesis process and schematic molecular structure of HGP are illustrated in Figure 10.

### 4.4. Characterization Methods of HGP

For the convenience of characterization, a portion of the HGP dispersion emulsion was dried, and the dried product was washed with ethanol to obtain solid HGP samples.

1. Fourier Transform Infrared Spectroscopy (FTIR): The samples were prepared using the KBr pellet method. The FTIR spectrum of HGP was recorded in the range of 4000–400 cm^−1^ using a Shimadzu IRTracer-100 Fourier transform infrared spectrometer (Shimadzu Corporation, Kyoto, Japan).
2. X-ray Photoelectron Spectroscopy (XPS): The surface chemical composition of the solid HGP samples was analyzed using an ESCALAB 250Xi X-ray photoelectron spectrometer (Thermo Fisher Scientific Inc., Waltham, MA, USA). The high-resolution C 1s and O 1s spectra were further fitted and analyzed.
3. Proton Nuclear Magnetic Resonance Spectroscopy (^1^H NMR): The ^1^H NMR spectra of the solid HGP samples were obtained using a JEOL NM-ECZL G 600 nuclear magnetic resonance spectrometer (JEOL Ltd., Akishima, Tokyo, Japan). The measurements were performed in CP/MAS mode with a resonance frequency of 600 MHz.
4. Thermogravimetric Analysis (TG/DTG): Thermogravimetric analysis of HGP samples was performed using a TGA/DSC 3+ thermal analyzer (Mettler-Toledo International Inc., Greifensee, Switzerland). The samples were heated from 40 to 800 °C under a nitrogen atmosphere at a heating rate of 5 °C/min, and the corresponding TG/DTG curves were obtained.
5. Scanning Electron Microscopy (SEM): The morphology of different samples was characterized using a SEM5000X scanning electron microscope (Guoyi Quantum (Hefei) Technology Co., Ltd., Hefei, Anhui, China). For the observation of HGP microspheres, the HGP dispersion emulsion was ultrasonically diluted with deionized water, dropped onto conductive adhesive, dried, and sputter-coated with gold before SEM observation. The microstructures of filter cakes and rock cores were observed after drying and gold sputter coating.
6. Transmission Electron Microscopy (TEM): The HGP dispersion emulsion was ultrasonically diluted with deionized water, and TEM images were obtained using a JEM-2100Plus transmission electron microscope (JEOL Ltd., Akishima, Tokyo, Japan). The samples were deposited on copper grids coated with a conventional carbon film for observation.

### 4.5. Analysis of the Impact of HGP on Drilling Fluid

The basic fluid was prepared by adding 4 wt% drilling fluid bentonite and 0.3 wt% Na_2_CO_3_ into deionized water, followed by low-speed stirring for 24 h. According to the experimental requirements, different amounts and types of plugging agents (SiO_2_ is added according to the mass of solid, and NF-1 and HGP are added according to the mass of lotion), as well as various concentrations of NaCl and CaCl_2_, were added into the basic fluid and stirred at high speed for 20 min to obtain the test drilling fluids. The prepared drilling fluids were then aged at different temperatures for 16 h according to the testing requirements. The drilling fluid performance tests were conducted using a ZNN-D6B six-speed rotational viscometer, ZNS-2A medium-pressure filter press, PPA sand disc plugging and filtration tester, and FA visual sand bed non-invasion filtration tester, all provided by Qingdao Xusheng Petroleum Instrument Co., Ltd., Qingdao, Shandong, China.

In order to ensure the objectivity of the test and eliminate the influence of accidental factors on the experimental results, performance tests were conducted three times according to relevant testing standards. The average of the results was taken as the representative data, and error bars were plotted based on the error range of each group of data.

1. The apparent viscosity, plastic viscosity, and API fluid loss of the drilling fluids were measured according to API standard procedures [42].
2. For the sand bed plugging test, quartz sand with a mesh size of 100–150 mesh was used. Ceramic sand discs with pore sizes of 1 μm, 3 μm, 5 μm, and 10 μm were selected for the sand disc filtration test. The testing procedures were performed according to API standards and the Chinese standard GB/T 16783.1-2025 *Petroleum and Natural Gas Industries—Field Testing of Drilling Fluids—Part 1: Water-based Drilling Fluids* [43]. The pressure difference for the sand bed test was set at 0.69 MPa, with a test duration of 30 min. The sand disc filtration test was conducted at 240 °C under a pressure difference of 3.5 MPa.

### 4.6. Analysis of the Enhancement Mechanism of HGP on Drilling Fluid

1. Particle size analysis: The particle size distributions of different drilling fluids and plugging agent dispersions were analyzed using a Mastersizer 3000 laser diffraction particle size analyzer (Malvern Panalytical Ltd., Malvern, Worcestershire, UK.). The median particle size (Dx50) was calculated and statistically analyzed separately.
2. Zeta potential analysis: The Zeta potentials of the samples were measured using a Malvern Zetasizer Nano Z nanoparticle size and zeta potential analyzer (Malvern Panalytical Ltd., Malvern, Worcestershire, UK). The zeta potential was determined by laser Doppler microelectrophoresis.
3. Mud cake permeability: The API mud cakes were first obtained through filtration tests. Subsequently, the drilling fluids were replaced with deionized water, and the mud cake permeability was calculated according to Darcy’s law (Equation (1)). The filtration rate was calculated as the average filtration volume within 30 min. The mud cake thickness was measured using a mud cake thickness and toughness automatic matching analyzer (ZN-1L, Qingdao Tongchun Petroleum Instrument Co., Ltd., Qingdao, Shandong, China). The viscosity of deionized water was taken as 1 mPa·s, the differential pressure was 6.9 × 10^5^ Pa, and the filtration area was 45.8 cm^2^.

(1) $$K_{c}=\frac{\mu t_{c}q}{\Delta PA}$$

where *K_c_*—permeability, mD;

*µ*—filtrate viscosity, mPa·s;

*t_c_*—mud cake thickness, cm;

*q*—filtration rate, cm^3^·s^−1^;

Δ*P*—differential pressure, 10^5^ Pa;

*A*—filtration area, cm^2^.

### 4.7. Analysis of HGP Blocking Ability and Mechanism

Pre-prepared 35 wt% NaCl and 5 wt% CaCl_2_ aqueous solutions were used as the dispersion media. The plugging agents were added into the solutions, followed by ultrasonic stirring for 10 min to obtain the plugging agent dispersions.

1. Microporous membrane plugging analysis: PTFE microporous membranes with pore sizes of 100 nm, 300 nm, 500 nm, and 1000 nm were selected to replace the filter paper used in the API filtration test, and the filtrate volume was continuously recorded during the filtration process. The pore size distributions of the PTFE microporous membranes before and after HGP plugging were analyzed using an iPore900 automatic membrane pore size analyzer (LiHuaLianKe (Beijing) Instrument Technology Co., Ltd., Beijing, China).
2. Core plugging analysis: Different plugging agent dispersions were aged at 240 °C for 16 h and subsequently injected into artificial sandstone cores with an initial permeability of 10 mD using an LDY50-180A core flow apparatus (Jiangsu Hongbo Gas Equipment Technology Group Co., Ltd., Dongtai, Jiangsu, China.) for plugging evaluation. The displacement pressure was 3.5 MPa, the confining pressure was 5 MPa, and the plugging time was 30 min [44]. The core plugging rate was measured according to the following procedure: the initial permeability of the core before plugging was measured using standard brine and recorded as *K*_1_. After the plugging experiment, the permeability of the core was measured again using standard brine and recorded as *K*_2_. The core plugging rate *R* was calculated according to Equation (2):

(2) $$R=\left(1-\frac{K_{2}}{K_{1}}\right)\times 100\%$$

3. High-temperature and high-pressure in-situ pressure transmission test: The in-situ pressure transmission test of the plugged core was conducted at 240 °C using a high-temperature and high-salinity plugging agent evaluation system developed by the Research Institute of Petroleum Engineering Technology, PetroChina. The displacement pressure was 3.5 MPa and the confining pressure was 5 MPa. The pressure variations at the inlet and outlet of the core holder were continuously monitored, and the experiment was terminated when the inlet and outlet pressures reached equilibrium.
4. Nuclear magnetic resonance (NMR) analysis: The T_2_ relaxation spectra and magnetic resonance imaging of the cores before and after plugging were analyzed using a MacroMR12-110H-I nuclear magnetic resonance analysis system (Suzhou Niumag Analytical Instrument Co., Ltd., Suzhou, Jiangsu, China).

## Data Availability

The data presented in this study are available on request from the corresponding author.
